# Mitochondrial Alterations, Oxidative Stress, and Therapeutic Implications in Alzheimer’s Disease: A Narrative Review

**DOI:** 10.3390/cells14030229

**Published:** 2025-02-06

**Authors:** Erica Spina, Riccardo Rocco Ferrari, Elisa Pellegrini, Mauro Colombo, Tino Emanuele Poloni, Antonio Guaita, Annalisa Davin

**Affiliations:** 1 Laboratory of Neurobiology and Neurogenetics, Golgi Cenci Foundation, Corso San Martino 10, 20081 Abbiategrasso, Italy; e.spina@golgicenci.it (E.S.); e.pellegrini@golgicenci.it (E.P.); m.colombo@golgicenci.it (M.C.); a.guaita@golgicenci.it (A.G.); a.davin@golgicenci.it (A.D.); 2 Department of Brain and Behavioral Sciences, University of Pavia, Viale Golgi 19, 27100 Pavia, Italy; 3 Department of Neurology and Neuropathology, Golgi Cenci Foundation, Corso San Martino 10, 20081 Abbiategrasso, Italy; e.poloni@golgicenci.it

**Keywords:** Alzheimer’s disease, mitochondria, mitochondrial dysfunction, aging

## Abstract

The relationship between aging, mitochondrial dysfunction, neurodegeneration, and the onset of Alzheimer’s disease (AD) is a complex area of study. Aging is the primary risk factor for AD, and it is associated with a decline in mitochondrial function. This mitochondrial dysfunction is believed to contribute to the neurodegenerative processes observed in AD. Neurodegeneration in AD is characterized by the progressive loss of synapses and neurons, particularly in regions of the brain involved in memory and cognition. It is hypothesized that mitochondrial dysfunction plays a pivotal role by disrupting cellular energy metabolism and increasing the production of reactive oxygen species (ROS), which can damage cellular components and exacerbate neuronal loss. Despite extensive research, the precise molecular pathways linking mitochondrial dysfunction to AD pathology are not fully understood. Various hypotheses have been proposed, including the mitochondrial cascade hypothesis, which suggests that mitochondrial dysfunction is an early event in AD pathogenesis that triggers a cascade of cellular events leading to neurodegeneration. With this narrative review, we aim to summarize some specific issues in the literature on mitochondria and their involvement in AD onset, with a focus on the development of therapeutical strategies targeting the mitochondria environment and their potential application for the treatment of AD itself.

## 1. Introduction

**Figure 1:**
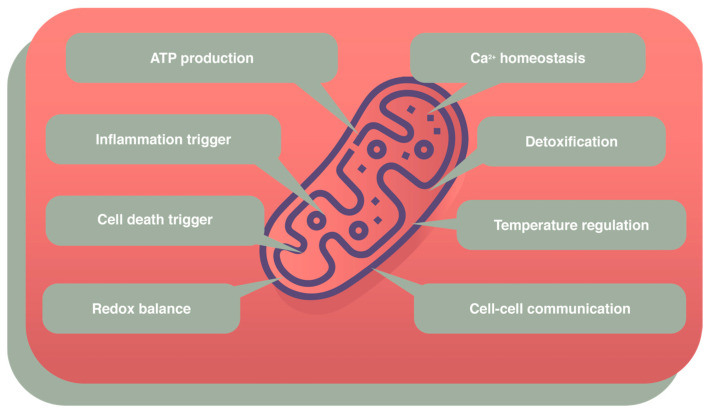
Schematic representation of the principal mitochondrial functions.

Mitochondria are cellular organelles with their own DNA and are responsible for the energetic requirement of the cell in the form of ATP molecules [1]. They are composed of two membranes, different in structure, functions, and permeability, and separated by the intermembrane space, between the outer mitochondrial membrane (OMM) and the inner mitochondrial membrane (IMM), which is the site where oxidative phosphorylation (OXPHOS) take place [2]. Mitochondria are not only the powerhouses of the cell but can also regulate temperature, maintain redox balance, and trigger inflammation and cell death, communication between brain cells, and detoxification [3,4]. Furthermore, mitochondria are involved in the balance, homeostasis, and signaling of calcium, the interaction of mitochondria with presynaptic and postsynaptic sites, and the production of ATP, ATP/ADP carriers, phosphate carriers, ATP synthases, and many other aspects of their own internal environment (Figure 1).

However, with aging, they are susceptible to progressive deterioration due to many intertwined mechanisms including the accumulation of mtDNA mutations, deficient proteostasis leading to the destabilization of respiratory chain complexes, reduced turnover of the organelle, and changes in mitochondrial dynamics [3]. This condition compromises cellular bioenergetics, triggers the accidental permeabilization of mitochondrial membranes causing inflammation and cell death, and enhances the production of ROS [3,5].

Particularly, a certain quantity of ROS is generated during the reaction catalyzed by the oxidative phosphorylation (OXPHOS) enzymes and their concentrations are under the regulation of dedicated enzymatic systems, which convert them into non-toxic elements [6,7]. Notably, under physiological levels, these molecules act as second messengers regulating several pathways, including cell death, proliferation, and senescence [6,7], and have a protective role for several mitochondrial functions, mitochondrial metabolism, dynamics, and cristae remodeling [7,8,9]. However, an overproduction of reactive species, together with a weakening of the cellular antioxidative defenses, may induce a condition named oxidative stress, which is involved in several pathological conditions, including neurodegeneration. Since a physiological decrease in antioxidant defenses occurs in older people, they are primarily prone to develop neurodegenerative diseases, including cognitive disorders [7].

Indeed, older age is the strongest risk factor for dementia [10,11] and is mainly responsible for all dementia conditions is Alzheimer’s disease (AD), a progressive neurodegenerative disorder [12], which is distinguished from the others with the presence of two neuropathological markers, the β-amyloid (Aβ) plaques and the tau neurofibrillary tangles, which are detectable in post-mortem brains [12]. Aβ plaques are composed of aggregates of Aβ peptides deriving from amyloid precursor protein (APP) cleavage by enzymes named secretases [12]: notably, the γ secretase generates the peptides named β-40, the soluble form, and β-42, the insoluble one, which aggregates and originates the characteristic plaques. Tau is a microtubule-associated protein responsible for the polymerization and stabilization of microtubules, and in turn, for the maintenance of the integrity of the cytoskeleton [12]. Neurofibrillary tangles are the result of an improper aggregation of a hyperphosphorylated tau which has a reduced affinity for the binding domain on the microtubules and is therefore deposited in the cytosol of the cells compromising cytoskeleton stability [12,13,14]. The combination of these abnormal complexes, Aβ plaques, and tau neurofibrillary tangles forms the neuritic plaque, which is the neuropathological hallmark of AD [12,13]. It is still a matter of discussion whether it is oxidative stress that determines the appearance of pathological protein aggregates or vice versa. Probably, both possibilities are true, and a vicious circle is generated and interferes with physiological brain functions, such as neuronal signal transmission, axonal transport, and synaptic activity leading to neurodegeneration [12].

In the past, the “mitochondrial cascade hypothesis” [15] was the most likely mechanism proposed to interpret how mitochondrial progressive impairment would lead to neurodegeneration and related neurodegenerative diseases. Nowadays, this theory has been gradually abandoned [16], and the possibility that mitochondrial impairment is not the direct cause of AD but is an early event seems to be more solid. It appears to be more likely that the presence of a pathological environment—consisting of increased ROS, accumulation of altered proteins, and mtDNA mutations—may lead to the accumulation of increasing cell damages including mitochondrial ones that, in turn, contribute to the progression of the disease [17]. Therefore, it is probable that a decrease in mitochondria functions, observed in the early stage of AD, makes the organelles themselves more sensitive to reactive species damage which exacerbates the initial damage. In other words, by imagining AD as a cliff, we can demonstrate that mitochondrial function may be related to resilience, and we could define the distance from this cliff and how quickly you approach it [18].

Recently, the role of Aβ plaques in exacerbating mitochondrial damage was proposed. It was proven that Aβ plaques interfere with the activity of two mitochondrial enzymes, α-ketoglutarate dehydrogenase and cytochrome c oxidase, since a lower expression was observed in the brains of AD patients [17]. Additionally, an increase in oxidative stress and mitochondrial dysfunction was observed in the presence of Aβ earlier than the characteristic symptoms associated with neuritic plaque pathology, further supporting the contribution of Aβ to mitochondrial damage [17].

In recent years, several studies investigated the involvement of mitochondria in AD to determine a relationship between their failure and the onset of the disease and possibly outline a specific sequence of events. These data include morphological studies of mitochondria, DNA damage related to oxidative stress, or metabolic alterations due to an impaired oxidative balance. The purpose of this review is to summarize the most significant available results correlating mitochondria, oxidative stress, and ATP production to AD, with a focus on results coming from human samples, including post-mortem brains as well as peripheral tissues. Following this, we present possible therapeutic and non-therapeutic approaches that are currently available and that could lead to an improvement in mitochondrial function by targeting these specific mechanisms and counteracting oxidative stress.

## 2. Defective Autophagy and Mitophagy in AD

In this review, we describe the presence of mitochondrial dysfunction, abnormal mitochondrial dynamics, and increased levels of oxidative stress and related damage in AD. This scenario is, however, accompanied by defective autophagy and mitophagy [19,20,21]. Autophagy is a lysosome-mediated and self-degrative survival process that allows the selective degradation of dysfunctional organelles and proteins. If this process is disrupted, cells tend to accumulate defects and abnormal mitochondria with an increase in oxidative stress, reduced cell availability, and accelerated aging [22]. Mitophagy specifically concerns mitochondria, and many factors may trigger mitophagy within a cell; most of this concerns mitochondria in terms of the release of cytochrome c, increased ROS production, and the opening of the mitochondrial permeability transition pore [23].

There are three defined types of autophagy: macro-autophagy, micro-autophagy, and chaperone-mediated autophagy. Macro-autophagy delivers cytoplasmic cargo to the lysosome through the intermediary of a double-membrane-bound vesicle, referred to as an autophagosome, that fuses with the lysosome to form the autolysosome. In micro-autophagy, by contrast, cytosolic components are directly taken up by the lysosome itself through the invagination of its membrane. Both macro- and micro-autophagy are also able to engulf large structures through both selective and non-selective mechanisms. In chaperone-mediated autophagy (CMA), targeted proteins are translocated across the lysosomal membrane in a complex with chaperone proteins (such as Hsc-70) that are recognized by the lysosomal membrane receptor lysosomal-associated membrane protein 2A (LAMP-2A), resulting in their unfolding and degradation [24].

Selective autophagy is crucial to destroy specific structures, organelles, or aggregate proteins. Mitophagy is a selective autophagy pathway that specifically promotes the turnover of mitochondria and prevents the accumulation of dysfunctional mitochondria which can lead to cellular degeneration. In mammals, it is mediated by two regulator pathways: PTEN-induced Putative Kinase 1 (PINK1)/Parkin and BNIP3/NIX.

The PINK1 and Parkin pathway is, so far, the best characterized. PINK1 is a kinase located in the inner mitochondrial membrane and is constantly degraded by mitochondrial proteins (matrix processing peptidase and presenilin-associated rhomboid-like) in healthy conditions [25]. Fragmented PINK1 is then translocated to the cytosol and further degraded. On the contrary, in damaged mitochondria, the depolarization of the mitochondrial membrane inactivates the proteins responsible for PINK1 degradation. Then, PINK1 can auto-phosphorylate, activating itself, and accumulate in the outer mitochondrial membrane to recruit cytosolic Parkin. Parkin is phosphorylated by PINK1 and enters the mitochondria, marking the mitochondria for autophagy. In this condition, Mitofusin 1 also becomes activated, leading to mitochondrial fission and facilitating mitophagy [26].

Other pathways can induce mitophagy such as the presence of NIX1 and BNIP3 which act as mitophagy receptors on the outer mitochondrial membrane surface. All these receptors contain sequences that bind LC3/GABARAP which can lead to the degradation of mitochondria [27].

Given these premises, the hypothesis of an association between Alzheimer’s disease and mitophagy defects dates to the 2000s [28]. After this, other studies supported the presence of a reduced mitophagic degradation in AD: Hu and colleagues showed, in AD brains, incremented levels of mitochondrial proteins COX IV and TOMM20 and an elevated ratio of mitochondrial DNA/nuclear DNA [29]. Furthermore, in hippocampi of sporadic AD, researchers observed an increase in PINK1 at early AD stages (Braak stage II and III) and in Parkin at later stages (Braak stage VI) with an increase in mitochondrial content markers in all pathologic stages [30], suggesting a reduced mitophagy linked to defects in the PINK1/Parkin cascade. Supporting this hypothesis, another study reported decreased PINK1 mRNA and protein in AD hippocampi (Braak stage V-VI) [31]. Other proteins involved in the mitophagic process were observed to be impaired in human AD brains [32] and also in the brains of ApoEε4 patients [33]. Fang et al. show a mitophagy reduction by 30–50% in AD hippocampi with an impairment in the initial steps of mitophagy (reduced mitochondrial LC3 recruitment and dysfunctional AMPK cascade), in addition to the accumulation of structurally and functionally damaged mitochondria [34]. In accordance with these results, an increase in the LC3-II/LC3-I ratio and p62, accompanied by a reduction in PINK1 and Parkin levels, was also uncovered in the mitochondria-enriched fraction of sporadic AD brains [35].

Other evidence of defective AD autophagy and mitophagy is also found in cells and fluids derived from patients. Skin fibroblasts of sporadic AD patients display a reduced formation of autophagic vesicles, a lower number of lysosomes, and an accumulation of TOMM20, as well as a Parkin level reduction, confirming dysfunctional autophagy and mitophagy [30]. Nevertheless, in fibroblasts of healthy people, it was observed that younger mitochondria were prevalently located in the periphery and the older surrounding nucleus. Differently, the fibroblasts of sporadic and familial AD patients reveal a different spatial organization, showing alterations in the mitochondrial transport and accumulation of dysfunctional mitochondria, enhanced LC3-II levels, and accumulations of p62 and TOMM20, reflecting an impaired lysosomal degradation ability [36,37].

Not least, the signature of an impairment in the mitophagy process was also reported in the peripheral studies of AD patients, showing a reduction in the autophagic factor ATG5 and a decrease in Parkin levels associated with an increase in PINK1 [38].

## 3. Impairment of Mitochondria Dynamics in AD Brains

Mitochondrial dynamics and the resulting overall morphology of the network are determined by fusion and fission events and by mitochondrial movement, both of which are highly dependent on the cell type and the functional state of mitochondria. In the Central Nervous System (CNS), mitochondria motility plays a crucial role, particularly in neurons where mitochondria move from the cell body, on the microtubules along the axon, to the synaptic site via anterograde transport to participate in neuronal activity or return to the cell body via retrograde transport to be degraded [9,39,40]. Therefore, it is not surprising that in neurons from AD brains whose microtubules’ integrity is impaired due to the presence of tau tangles, mitochondria distribution is negatively affected [8,17]. Indeed, in human post-mortem brains, a different mitochondria distribution is evident when comparing AD cases and healthy controls. Specifically for neurons isolated from the hippocampus, which is known to be one of the primary brain regions affected by this neurodegenerative condition, researchers reported the presence of a perinuclear ring of mitochondria with almost total absence in the distant processes, while a physiological distribution in synapses is maintained in healthy subjects [16]. Regarding astrocytes, it was observed that under physiological conditions, mitochondria are highly associated with glutamate transporters and localized in the fine terminal processes (leaflets) of astrocytes [41,42]. These structures surround synapses and contribute to the regulation of ionic homeostasis in synaptic transmission [43]. In oligodendrocytes, on the other hand, mitochondria are distributed and move through the myelin sheath [44]. Moreover, they show a reduced surface area of cristae, though the relationship between this feature and their function is still under investigation [45].

Mitochondria undergo physiological switching of their shape and size—from small and round (grain-like morphology) to giant and elongated (thread-like morphology)—via processes known as fusion and fission that often occur simultaneously. Fusion occurs when mitochondria membranes meet and merge into one, leading to the formation of a single enriched organelle with a better ability to generate ATP [8,46]. Fission determines an increase in mitochondria volume and the division of the mitochondria into two often not equally sized daughter mitochondria with the purpose of regulating some mechanisms such as apoptosis and mitophagy and responding to the alteration of cell bioenergetic requests [8,46].

The mitochondrial dynamics system is also known for regulating the response to various physiological challenges. For example, upon starvation, mitochondria acquire an elongated tubular structure that has been identified to promote cristae remodeling, mtDNA sharing, and delayed apoptotic signaling [47]. Additionally, modifications in mitochondrial ultrastructure are evident in various pathological conditions, including Alexander Disease—with a major involvement of astrocytes [48]—and multiple neurodegenerative disease models with a major involvement of microglial mitochondria [49].

Therefore, the correctness of these dynamics is fundamental for several cell processes such as energy metabolism, ROS generation, and the regulation of apoptosis [40]. These mechanisms depend on the activity of specific mitochondrial proteins named fission and fusion proteins. Particularly, fission requires some outer mitochondrial membrane proteins, including the GTPase dynamin-like protein 1 (DLP1) also named dynamin-related protein (DRP1), mitochondrial fission 1 protein (FIS1), and the mitochondrial fission factor (MFF) involved in the recruitment of DLP1 [40]. Fusion also depends on the activity of some GTPases, such as Mitofusin 1 (MFN1), Mitofusin 2 (MFN2), and Optic Atrophy 1 (OPA1) [40,46,50]. Alterations in mitochondria dynamics have been widely described in AD patients with effects on the cristae structure, the electron transport chain, and, consequently, on redox homeostasis and all the pathways dependent on its balance [16]. Several of these studies reported evidence of abnormal mitochondria morphology in some specific brain areas and human cell cultures, probably due to impaired dynamics. Among them, Flannery and Trushina [9] found giant mitochondria they called mitochondria-on-a-string (MOAS) in the hippocampus and cortical brain of AD patients; García-Escudero and collaborators [40] reported data from sporadic AD patients indicating the presence of elongated and interconnected mitochondria in fibroblasts [40,51] and fragmented mitochondria in neurons, with predominant localization in the perinuclear space, probably as a result of mitochondrial fission protein FIS167 overexpression and a concurrent involvement of tau proteins [16,40]. Moreover, the most affected neurons present oversized mitochondria, in terms of length and width, indicating an imbalance of their dynamics in favor of the fission process, a phenotype probably dependent on an altered expression pattern of the proteins involved in fission and fusion mechanisms in AD patients compared to healthy controls [9,40]. Nevertheless, data are very controversial in different studies. For instance, in the frontal cortex of post-mortem AD brains at both early and severe stages, an increased expression of DRP1 and FIS1 and a decreased expression of MFN1, MFN2, OPA1, and mitochondrial import receptor subunit TOM40 (TOM40) were detected, leading to mitochondrial fragmentation [9]. Additionally, Wang X. and collaborators [51] observed reduced levels of the mitochondrial fission protein DLP1 in fibroblasts with an altered mitochondrial distribution. Nevertheless, these differences may be due to the variety of samples used among all the studies and to the different stages of pathology of the affected donors included in the distinct studies.

## 4. The Role of Mitochondrial Proteins in AD

Mitochondrial dysfunction leads to reduced ATP production, impairing essential neuronal and glial functions and contributing to cognitive decline [52,53]. The accumulation of Aβ, a hallmark of AD, has been linked to mitochondrial dysfunction [54]. Aβ can directly interact with mitochondrial proteins, disrupting their function and leading to increased oxidative stress and impaired energy production. Studies on animal models have shown that Aβ is present within the mitochondria of APP/PS1 transgenic mice at an early stage, particularly in synaptic mitochondria, an aspect that correlates with synaptic damage and that could contribute to cognitive decline [55,56].

Several mitochondrial proteins have been implicated in AD pathogenesis, with a particular focus on those involved in the electron transport chain (ETC). The ETC is responsible for the majority of ATP production in mitochondria. Dysregulation of ETC complexes, particularly Complex I, has been consistently observed in AD [57]. One study utilizing iTRAQ quantitative proteomics found that subunits of Complex I, including NDUFA4 and NDUFA9, were significantly down-regulated in early-onset AD patients. These subunits are crucial for the stability and function of Complex I, and their down-regulation suggests a potential defect in the junction between the membrane and matrix arms of the complex. This destabilization could impact electron transport, leading to reduced ATP production and increased oxidative stress [57].

Other ETC complexes have also been found to be affected in AD. Subunits of Complex II (SDHA and SDHD), Complex III (UQCRC1 and UQCRC2), and Complex V (ATP5B, ATP5H, ATP5I, and ATP5J) were down-regulated in early-onset AD patients. These alterations further contribute to the overall impairment of mitochondrial function and energy production [57].

In addition to ETC complexes, other mitochondrial proteins played a role in AD. The *ABAD* gene, for instance, encodes for the 3-hydroxyacyl-CoA dehydrogenase type II, a protein in the mitochondrial matrix that was observed to interact with Aβ and promote oxidative stress [58].

It was also observed that the mitochondrial ATP synthase subunit can bind to the extracellular domain of APP and Aβ [59]. Moreover, Aβ interacts with mitochondrial matrix components contributing to mitochondrial dysfunction and impaired ATP production [59,60].

In the context of proteostasis, Aβ interacts with the mitochondrial protease HTRA2, potentially altering its activity and contributing to AD pathogenesis. HTRA2 is involved in protein quality control and apoptosis, and its dysregulation has been linked to mitochondrial dysfunction and neuronal death [61]. Furthermore, the mitochondrial chaperone protein mtHsp70, also known as Mortalin, crucial for protein folding and protection against oxidative stress, has been shown to be decreased in AD [62].

An AD-specific alteration was observed also for VDAC1, a voltage-dependent anion channel protein located in the outer mitochondrial membrane. Increased VDAC1 blocks mitochondrial permeability transition pores, disrupting the transport of mitochondrial proteins and metabolites. It also compromises VDAC gating, leading to defects in oxidative phosphorylation [63,64,65].

These findings suggest that mitochondrial dysfunction, driven by Aβ accumulation and the dysregulation of key mitochondrial proteins, plays a critical role in the pathogenesis of AD. Further research is needed to fully elucidate the complex interplay of these proteins and to develop targeted therapies aimed at mitigating mitochondrial dysfunction in AD.

## 5. Mutations in mtDNA and Oxidative Stress-Dependent Alterations of DNA Damage Repair Mechanisms

Human mitochondrial DNA (mtDNA) is a single circular molecule of 16.5 Kb present in multiple copies in a single mitochondrion according to the energy requirement specific to the cell type. mtDNA is composed of two strands, the heavy and the light strand, and contains 37 genes, including 2 encoding ribosomal RNA, 22 tRNA, and 13 encoding proteins required for the OXPHOS [3]. It is prone to a higher rate of mutation and damage compared to nuclear DNA because of a series of intrinsic characteristics, such as the absence of histone proteins, the higher rate of replication, and concurrently lower efficiency of the DNA repair mechanisms: it was demonstrated that mitochondria lack a nucleotide excision repair (NER) pathway, but they have a robust base excision repair (BER) pathway, using isoforms of many of the BER enzymes in the nucleus, which are involved in the repair of oxidative lesions [66,67]. For all these aspects, during the lifespan, mitochondria accumulate mutations on mtDNA that may contribute to neurodegeneration, which may also be due to the specific susceptibility of neurons to this type of damage since they are post-mitotic cells [7]. Focusing on AD, several studies detected alterations in samples from affected people compared to healthy controls, and some examples are reported below. In summary, they come from analysis performed on cerebrospinal fluid (CSF) and blood, or post-mortem brains, and the types of impairment include copy number variations, in terms of a number of mtDNA molecules, as well as mutations, deletions, and alterations of the DNA structure. Indeed, under cell damage conditions, mtDNA can be released from the cell both as circulating cell-free mtDNA (cf-mtDNA) or inside vesicles [68]. However, a significant increase in ROS, occurring in such pathological conditions including AD, may induce the release of mtDNA fragments through large pores on the cell membrane [66,68]. Additionally, these circulating fragments can be recognized by the immune system as damage-associated molecular patterns (DAMPs) or antigens, a signal that activates a proinflammatory response [68]. These fragments are detectable in plasma and CSF and therefore represent a potential biomarker for AD. Nevertheless, data collected so far are very controversial, since some studies report an increase in free mtDNA in patients with a faster progression of the disease, compared to controls, while other authors observed an increase in AD patients, but a non-significant difference was observed between affected and control groups. The most suitable explanation for this variable trend lies in the different susceptibility levels among the various types of neurons constituting a single brain region. Specifically, a pronounced increase in mtDNA levels in the most vulnerable hippocampal and cortical neurons, such as pyramidal cells, was observed [68].

Because mtDNA is present in multiple copies per cell, then copy number variation in mtDNA (mtDNAcn) was suggested as a suitable marker of mitochondrial abundance [69]. It correlates with the energetic request and metabolic state of the cell, and, as a result, its alteration may be a signal of mitochondrial damage. In support of these data, some studies observed a decrease in mtDNAcn in post-mortem AD brains compared to healthy controls in different cerebral areas, such as the hippocampus, prefrontal and frontal cortex, and posterior cingulate cortex [68]. Additionally, differences in mtDNAcn are evident among cells from different brain areas [68].

A higher mutation rate on mtDNA was detected in AD patients compared to healthy controls, and it occurs in genes encoding some subunits of the enzymatic complexes of the respiratory chains, such as the catalytic subunit of cytochrome c oxidase (CO), and subunits 1 and 2 of the NADH dehydrogenase, control regions, tRNA, and the polymerase γ [68]. These mutations determine a decrease in respiratory chain activity and then ATP production with a parallel increase in reactive species in AD patients [16]. However, differences between distinct haplogroups were observed, so some haplogroups with a specific combination of Single-Nucleotide Polymorphisms (SNPs) in mtDNA seem to be particularly susceptible to AD compared to others, but the results are still controversial [70].

Redox dysregulation and oxidative stress are among the main factors responsible for both nuclear DNA (nDNA) and mtDNA damage by inducing structural alterations, documented in several neurodegenerative conditions, that count single- and double-strand breaks as well as oxidative stress-induced DNA damage, observed in the brain tissue, including the parietal and temporal lobes, and peripheral blood. Particularly, Mecocci P. and colleagues revealed three-fold more oxidative damage in mtDNA in AD patients [71]. Specifically, higher levels of 8-hydroxy-2′-deoxyguanosine (8-OHdG) and 8-hydroxyguanosine (8-OHG) were reported in AD brains compared to age-matched control samples [71], and 8-hydroxyadenine (8-OHA) and 5-hydroxy cytosine were observed to be more frequent in AD patients compared to healthy controls. Concurrently, an impairment of the DNA repair pathways was observed in the brains of AD patients [7]. For instance, mutations on 8-oxoG glycosylase (OGG1), the enzyme involved in the repair of the gene 8-oxoG lesions occurring in nDNA and mtDNA, as well as on MRE11, involved in DNA double-strand break damage, were observed in the brains of AD patients compared to healthy controls. Impairment of DNA repair mechanisms contributes to the further accumulation of DNA damage and then to neurodegeneration [7]. An impairment is also evident in some genes involved in the oxidative imbalance, such as *SOD1* and *SOD2* encoding superoxide dismutase, an antioxidant enzyme located, respectively, in the cytoplasm and mitochondria that converts the superoxide radicals to molecules of oxygen and hydrogen peroxide [7]. The decrease in DNA repair mechanisms and the antioxidant defenses occurring in older people could be the reason why aging itself is the main risk factor for neurodegeneration [7].

Mitochondrial defects may also be a secondary response to nuclear DNA repair defects: for instance, mutations in the *XPA* gene, encoding the DNA damage binding protein *XPA*, can lead to defective mitophagy even if XPA proteins are present in the nucleus and not in the mitochondria, indicating the existence of signaling from nucleus to mitochondria [72].

Further, the down-regulation of DNA polymerase β (pol β) in AD brains has also been demonstrated: this protein is involved in the BER pathway, and its reduction is already present in early disease stages, further declining with disease progression [72]. Pol β knockout cells show hypersensitivity to DNA damage and mitochondrial dysfunction [73].

There is another concept that is important to consider: mitochondria within each cell may vary from each other considering their mitochondrial genome profile. This concept is known as DNA heteroplasmy and this implies that mtDNA mutations can affect all copies of the mtDNA in a cell (termed homoplasmy) or only a fraction of the mtDNA molecules (termed heteroplasmy). Increased levels of heteroplasmic mtDNA deletions as well as heteroplasmic point mutations were found in the brains of AD patients [74,75], leading to the hypothesis that pathogenic mtDNA mutations, when they exceed certain thresholds, could contribute to the mitochondrial dysfunction observed in late-onset neurodegenerative diseases. However, by contrast, a recent high-throughput sequencing study looking at heteroplasmic point mutations found no evidence for an association with AD [76]. On this matter, further investigations are necessary to expand on the real contribution that these accumulated mtDNA mutations can present to the neurodegenerative AD process, also considering that, as reported by Shang et al., mtDNA mutations can also be found in physiological aging; thus, they cannot be considered a direct cause of AD pathogenesis [77]. An exhaustive list of mtDNA alterations is reported by the same authors in their review [77].

## 6. Role of γ-Secretase on Mitochondria Function

The role of the γ-secretase complex on the pathogenesis of AD is widely demonstrated, since the accumulation of the Aβ peptide, which is the component of the amyloid plaques, is due to impaired activity in the cleavage of the APP carried out by the γ-secretase itself [16]. This is also corroborated by the existence of familiar forms of AD resulting from mutations in the APP gene, as well as in presenilin 1 (PS1 or PSEN1) and presenilin 2 (PS2 or PSEN2) genes, coding for these two proteins participating in the γ-secretase complex which are essential for its activity [16,46]. It was suggested that an altered activity of γ-secretase and, in turn, an incorrect processing of APP may also be responsible for the abnormal mitochondria distribution that is often visible in cells from AD brains, as discussed before. However, the relationship among these dysregulations is still controversial and not completely clarified [16]. Among the evidence supporting the existence of a possible correlation between mitochondria and the γ-secretase pathway, there is the presence of some elements of the γ-secretase complex in these organelles, such as presenilin 1 [78]. Additionally, it was observed that APP accumulates in mitochondria import channels so it was proposed that part of the Aβ peptide produced in this region may contribute to the impaired mitochondria activity observed in AD patients [79]. Despite this evidence, another group reported that γ-secretase concentration in mitochondria is very low compared to the other membranes where it usually accumulates, such as the cell and the endoplasmic reticulum. Additionally, it is known that the γ-secretase complex is active only in the lipid raft regions that are absent in the mitochondrial membrane. According to Gomez and coworkers, these observations are in contrast with the hypothesis that mitochondria impairment may be responsible for the early pathogenesis of familial AD due to γ-secretase mutations [16].

## 7. Metabolic Alterations Observed in AD Patients Related to Mitochondrial Activity

Oxidative stress is also responsible for several metabolic pathway impairments that participate in AD onset and progression. These changes in metabolism may be detected following some metabolites in the CSF as well as in the plasma, providing potential therapeutical and early diagnostical targets when they demonstrate correlation with the pathology [80]. Data from AD patients’ samples reveal an impairment of lipid metabolism [80,81] since it is well known that an increase in reactive species directly degrades lipid molecules. Consistently, a significant reduction in lipid molecules in the inferior temporal and middle frontal gyrus of AD patients compared to healthy controls was observed [80].

Glucose metabolism was also revealed to be affected by ROS increase, supported by an increase in lactate concentration in AD patients’ CSF compared to healthy controls [81]. Cerebral glucose metabolism can be impaired by the dysfunction of intracellular oxidative catabolism or the abnormality of glucose transportation due to insulin resistance [82], a condition of reduced responsiveness of insulin-targeting tissues to physiological levels of insulin [83]. AD patients show decreased GLUT1 and GLUT3 expressions [4] and glucose transporters are mainly expressed in astrocytes and neurons, respectively [82], leading to reduced glucose uptake [4]. Glucose hypometabolism is indeed a common feature of AD at clinical stages, indicating the loss of neuronal function in specific brain regions [81]. Furthermore, an increase in pyruvate concentration in the CSF of AD patients compared to healthy controls was reported, sustaining an impairment of tricarboxylic acid metabolism [80]. Oxidative stress is also closely related to neuroinflammation [84,85], and the alteration of mitochondrial dynamics in microglial cells can affect proinflammatory mediator expression [84,86]: microglia-mediated neuroinflammation is a main feature of many neurodegenerative diseases, including AD [84,85]. Mitochondrial dysfunction also results in calcium dyshomeostasis [87] and decreased adenosine triphosphate (ATP) and phosphocreatine (PCr) in AD patients [84,88]: creatine, including PCr, is an important energy shuttle that also serves as an intracellular buffer for ATP by providing a readily available resource of high-energy phosphate through the creatine kinase reaction [84]. Additionally, NAD^+^ levels are lower in post-mortem AD brains and can be linked to the impairment of the NAD^+^–mitophagy axis and the DNA repair pathway [72].

## 8. Therapeutical Strategies Aiming to Prevent and/or Restore Mitochondria Impairment

**Figure 2:**
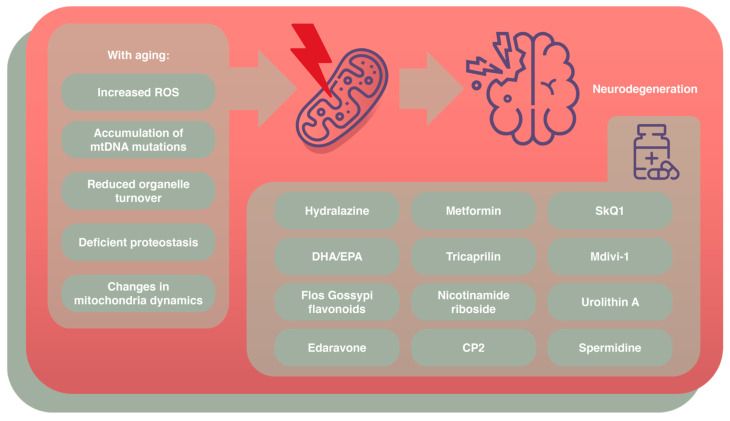
A schematic representation of the main aging-related alterations linked to mitochondrial damage and neurodegeneration and the pharmacological therapies designed to contrast this mechanism.

To date, there are no therapeutical molecules able to directly contrast the progression of AD or restore the lost cognitive functions. Traditionally, the research focused on AD-typical proteinopathies (Aβ plaques and neurofibrillary tangles) to eliminate them [89]. Specifically, the approved anti-amyloid treatments target Aβ peptides by inhibiting their aggregation with small molecules or activating a microglial response of phagocytosis induced by monoclonal antibodies (mAbs) [89]. Among the latter, aducanumab, lecanemab, and donanemab were the mAbs approved by the FDA for the treatment of early-stage AD patients with amyloid presence verified via amyloid imaging by positron emission tomography (PET) or CSF investigation [90]. Unfortunately, there was no significant clinical gain, despite the reduction in Aβ plaques and the improvement of typical AD biomarkers in phase 2 and phase 3 clinical trials [91]. Additionally, recent MRI studies reported severe adverse effects in patients treated with some antibodies approved for clinical trials, including brain edema and hemorrhage, named Amyloid-Related Imaging Abnormalities (ARIAs), which may be responsible for severe clinical manifestations such as seizures, confusion, nausea, headache, and death, although rarely [92]. Probably, the Aβ plaques and the tau neurofibrillary tangles are the final and irreversible manifestation of earlier mechanisms. For all these reasons, and considering that aging itself is characterized by systemic biological impairment, several other different targets are under investigation, such as neuroinflammation, ApoE, lipids and lipoprotein receptors, bioenergetics and metabolism, synapses, and mitochondrial and oxidative stress [90]. Concerning the latter, a variety of biomarkers of mitochondrial damage and oxidative stress were reported in different human samples, including post-mortem brains, peripherical cells, plasma, and CSF, comparing AD patients to healthy controls [17]. Therefore, several studies, in both preclinical and clinical phases, focus on identifying possible compounds capable of restoring mitochondrial loss of function and contrasting the damage derived from reactive species. Overall, it is possible to distinguish between pharmacological and non-pharmacological strategies whose aim is to prevent oxidative stress. The first ones include compounds likely able to restore a lost function or at least decelerate the progression of the disease (Figure 2), while the others mainly focus on lifestyles capable of preventing or delaying neurodegeneration by improving the oxidative functions of mitochondria [9].

### 8.1. Non-Pharmacological Strategies

Physical exercise, caloric restriction, and ketogenic diets were demonstrated to reduce or slow down the development of cognitive decline and the incidence of dementia [4,9,93]. The biological mechanisms responsible for these effects are not clear; however, they likely contribute to improving mitochondria activity. Both physical exercise and caloric restriction may induce higher turnover and biogenesis that, in turn, trigger a lower production of ROS and then lower oxidative stress levels. Additionally, it was observed that caloric restriction also acts on mitochondria dynamics, morphology, and function [9]. Concerning ketogenic diets, it was observed that ketone bodies improve mitochondria function and, concurrently, antioxidant defenses that, in turn, induce a decrease in ROS accumulation and oxidative stress. The improvement of mitochondria function likely depends on the energy provided by ketone bodies that would be an advantage in a condition of impaired glucose uptake such as the neurodegenerative environment. Moreover, it was demonstrated that a ketogenic diet determines a reduction in ROS production and an improvement of antioxidant defenses and mitochondria activity [9].

### 8.2. Pharmacological Strategies

Among the pharmacological strategies targeting mitochondria and oxidative stress, some compounds showed beneficial effects in preclinical studies, and at present, few of them are in phase 3 and phase 2 clinical trials [90] (Table 1). Hydralazine is able to activate antioxidant pathways and increase mitochondria function and then ATP production in low-to-moderate-AD subjects, and it is currently in a phase 3 trial [90], as well as eicosapentaenoic acid (EPA) and docosahexaenoic acid (DHA) polyunsaturated fatty acids [90,94,95]. Among the molecules in phase 2 trials, Flos Gossypii flavonoids showed antioxidant and anti-inflammatory effects, and they deserve to be mentioned together with Edaravone, which was capable of inhibiting Aβ aggregation and the related oxidation, reducing neuroinflammation and glia activation in preclinical studies; it is currently being tested on a group of early-stage AD subjects [90,95]. Additionally, it was observed that some molecules that have effects on the OXPHOS complexes, partially inhibiting them, seem to have a protective role against aging and cognitive disorders. It was suggested that this is due to an effect on mitochondria that is similar to the one observed in patients performing caloric restriction or physical exercise described above. For instance, among these compounds, metformin, a syntenic compound of guanosine used to treat Type II Diabetes Mellitus [96], was observed to reduce neuroinflammation and oxidative stress and concurrently improve mitochondria and brain function, and it is currently in a phase 2/3 clinical trial [9,90,95]. Therefore, it is not surprising that a reduced risk of cognitive impairment was observed in MCI and AD patients treated with metformin [9].

Another bioenergetic and metabolic agent is Tricaprilin, a caprylic triglyceride metabolized to ketones to stimulate mitochondria and provide an alternate neuronal metabolic pathway, improving brain metabolism in the setting of insulin resistance and glucose hypometabolism, and it is presently in a phase 3 clinical trial [90,95].

Mitochondrial function seems to also be improved by NAD^+^ boosters, such as nicotinamide riboside (NR), a proprietary form of nicotinamide or vitamin B3 with antioxidant activity, and it is also a very powerful mitophagy inducer: several clinical trials were assessed to evaluate its effect on brain, cognition, and oxidative stress both in MCI subjects and in AD patients (NCT02942888, NCT04078178, NCT04430517, NCT03482167, and NCT03061474) [90,95,97].

The tricyclic pyrone named CP2 seems to be a promising drug; it is a mitochondrial Complex I inhibitor, with a safety profile and low toxicity and with the capacity to also reduce, in stress conditions, proton leak and counteract Aβ aggregates [4,98].

Another potential medicine was SkQ, the first mitochondria-targeted medicine used in clinical practice [4]. It eliminates the ROS from the cells, and long-term treatments decrease the hyperphosphorylation of Aβ-42 and APP [99].

A new promising drug is mitochondrial division inhibitor-1 (Mdivi-1), a particular inhibitor of protein DRP-1, which is the mitochondrial element responsible for membrane fragmentation. This molecule interferes with different steps of mitochondria bioenergetics, reducing Aβ deposition [100,101]. As discussed above, an impairment in mitochondria dynamics is observed in AD patients; therefore, also targeting fission and fusion mechanisms was suggested as a novel possible therapeutic approach [8].

Urolithin A, which is derived from ellagitannins polyphenols, is able to improve mitochondrial homeostasis in nematodes and mammals via the stimulation of mitophagy. Other urolithin treatment-related effects are the reduction in A-beta pathology stimulating the phagocytosis of A-beta plaques, the reduction in microglial mitochondria damage, and a decrease in inflammation [34]. The first human clinical trial of urolithin (NCT02655393) was conducted in healthy sedentary elderly individuals, demonstrating its safety and benefits in the modulation of mitochondrial gene expression [102].

Spermidine, a small organic molecule, stimulates the PINK1/Parkin mitophagy pathway in human fibroblast, and in the clinical trial NCT02755246, spermidine improved hippocampal-dependent memory in elderly subjects with MCI [103].

Another intriguing aspect of cellular physiology is that mitochondria can be transferred. To date, it is known that mitochondria transfer can occur in three different ways: (1) transient cellular connections from one cell to another; (2) transfer via extracellular vesicles; and (3) the release of free mitochondria [104]. In the first case, transient intercellular connections are formed via connexin 43 (Cx43) gap junctional channels or tunneling nanotubes that are responsible for the mitochondria transfer along actin microtubules using the mitochondrial Rho GTPase 1 [105]. Despite this, the reasons that lead to the selective transfer of mitochondria rather than other cellular components are currently under investigation. Transfer via extracellular vesicles can occur in different ways according to the cell of origin and the biological purpose. Brown adipocytes and osteoblasts, for instance, release mitochondria-loaded vesicles to dispose of damaged or fragmented mitochondria [106,107], as well as cardiomyocytes [108,109]. Platelets or astrocytes, on the other hand, are able to release functional mitochondria-loaded vesicles in order to sustain, respectively, inflammation and leukocyte activation, and neuronal mitochondrial metabolism after ischemic stroke [110,111]. Cell-free mitochondria release, then, depends on mitochondrial fission proteins and the subsequent uptake during macropinocytosis [112], but the fate of these mitochondria in recipient cells is still unclear [104].

Considering all of these factors, it is clear that the mechanism of mitochondria transfer could represent an interesting target in order to develop novel therapeutical strategies, especially in the context of neurodegeneration and dementia considering the lack of effective approaches.

In this regard, promising results were obtained with several studies on animal models. In particular, it was observed that mitochondria injections can restore the motor skills of rats and mice, respectively, with induced brain ischemia and Parkinson’s disease [113,114]. Furthermore, intravenously injecting mitochondria from the livers of young mice into aged mice had a beneficial effect on motor and cognitive performance [115]. Considering this, and also considering the mitochondrial dysfunction inherent to aging and AD, investigating the biological role of mitochondrial transfer is essential in order to develop new mitochondrial transplantation strategies, whether xenogeneic or allogeneic. This could be an effective new solution to help address AD and other forms of neurodegeneration and cognitive decline.

**Table 1:** Pharmacological compounds and their biological effects.

Name	Class	Standard Application and Effect	Mitochondrial Environment and AD	Clinical Trials	References
Hydralazine	Hydrazinophthalazine	Hypertension and heart failure	Increased ATP production	Phase 3 (NCT04842552)	[90]
Eicosapentaenoic acid + Docosahexaenoic acid	Long-chain fatty acid	Inflammation and hypertriglyceridemia	Increased ATP production	Phase 3 (NCT03691519)	[90,94]
Docosahexaenoic acid	Long-chain fatty acid	Several beneficial effects on cardiovascular activity, cognition, and visual functions	Increased ATP production	Phase 2 (NCT03613844)	[90,94,95]
Flos Gossypii flavonoids	Flavonoid	Oxidative stress and inflammation	Reduced ROS production	Phase 2 (NCT05269173)	[90,95]
Edaravone	Pyrazolone	Antioxidant, free radical scavenger	Inhibited Aβ aggregation and attenuated Aβ-induced oxidation	Phase 2 (NCT05323812)	[90,95]
Metformin	Biguanide	Hyperglycemia	Improved mitochondrial function	Phase 3 (NCT04098666)	[9,90,95]
Tricaprilin	Caprylic triglyceride	Insulin resistance	Improved mitochondrial metabolism	Phase 3 (NCT05809908)	[90,95]
Nicotinamide riboside	Proprietary form of nicotinamide or vitamin B3	Antioxidant	Improved mitochondrial function	Phase 1 (NCT04430517) Not applicable (NCT02942888) Not applicable (NCT04078178) Phase 1 and 2 (NCT03482167) Phase 2 (NCT03061474)	[90,95,97]
CP2	Tricyclic pyrone	N/A	Inhibited mitochondrial Complex I and attenuated Aβ aggregation	N/A	[4,98]
SkQ1	Plastoquinol-based antioxidant	N/A	Reduced intracellular ROS	N/A	[4]
Mdivi-1	Quinazolinone	N/A	Inhibited mitochondrial division and reduced Aβ deposition	N/A	[100,101]
Urolithin A	Metabolite from ellagitannins polyphenols	Delays the development of age-related decline in muscle health	Stimulation of mitophagy and phagocytosis of Aβ plaques	Phase 1 (NCT02655393)	[34,102]
Spermidine	Aliphatic polyamine	Beneficial effects on cognition	Stimulation of mitophagy	Phase 2 (NCT02755246)	[103]

## 9. Conclusions

The contribution of mitochondria to Alzheimer’s disease is still controversial, with many uncertainties in giving it a role either as a trigger or as a driver of pathology. Regardless of the contradictory results of some studies, it is clear that mitochondria produce the energy necessary for the normal functioning of the synapses. Furthermore, AD can be considered a synaptopathy with hypometabolism and synaptic failure in the involved areas, which is associated with the breakdown of mitochondria. Therefore, it is difficult to question their central role in the pathogenesis of neurodegenerative diseases and AD in particular. Probably, genes have a core role in determining mitochondrial biology and resilience. The age-dependent changes in the mitochondrial function require a molecular and physiological response to preserve homeostasis, generating an adaptative response. However, it is possible that the change in some cases is so radical that successful compensation is no longer possible with the consequent onset of pathological aging. In this review, we have mainly focused on studies that describe the changes occurring in the mitochondria of human subjects affected by AD, particularly in the brain.

The mitochondrial cascade hypothesis suggests that effective treatments can improve and reinforce mitochondrial function also altering cell bioenergetics.

The study of therapies aimed at improving mitochondrial function is based on the consideration that any inadequate mitochondrial function, generated by the accumulation of toxic entities or conditions related to aging, causes a worsening of brain performance.

Therefore, addressing phenomena that even partially restore dysfunction could confer the greatest benefit.

Another important challenge is the development of new methods to transport drugs directly into the mitochondria and not into other regions of the body, where these same drugs could cause adverse reactions.

The function of mitochondria and its influence on biological processes goes far beyond what is already known since mitochondria are independent and have several specialized tasks in different tissues. Knowing more about their functions and how they can be modified in aging and neurodegeneration represents a compelling perspective.

## Data Availability

No new data were created or analyzed in this study.
